# Weight Constrained DEA Measurement of the Quality of Life in Spanish Municipalities in 2011

**DOI:** 10.1007/s11205-016-1426-y

**Published:** 2016-08-08

**Authors:** Eduardo González, Ana Cárcaba, Juan Ventura

**Affiliations:** University of Oviedo, Av. Cristo s/n, 33006 Oviedo, Spain

**Keywords:** Quality of life, Spain, Municipalities, DEA, Weight restrictions

## Abstract

This paper measures quality of life (QoL) in the 393 largest Spanish municipalities in 2011. We follow recent descriptions of QoL dimensions to propose an integrated framework composed of eight dimensions: material living conditions, health, education, environment, economic and physical safety, governance and political voice, social interaction, and personal activities. Using different sources of information we construct 16 indicators, two per each of the QoL dimensions considered. Weight constrained data envelopment analysis (DEA) is then used to estimate a composite indicator of the QoL of each municipality. Robustness is checked by altering the weight ranges introduced within the DEA specification. Results show that the Northern and Central regions in Spain attain the highest levels of QoL, while the Southern and Mediterranean regions report lower scores. These figures are consistent with those obtained by González et al. (*Soc Ind Res* 82:111–145 2011) for the Spanish municipalities in 2001, although both the sample and the indicators used are different. The analysis also shows that, while it is important to restrict weights in DEA, the specific restrictions used are less important, since all the composite indicators computed are highly correlated. The results also show important differences between per capita gross domestic product and QoL at the provincial level.

## Introduction

The progress and development of society should be the ultimate goal of public policy decision making. Social progress has been traditionally associated with economic macro-indicators, gross domestic product (GDP) being the most extended one.^1^ However, the creator of GDP, Simon Kuznets warned against the potential misuse of GPD as a measure of well-being: “the welfare of a nation can scarcely be inferred from a measurement of national income” (Kuznets 1934: 7). A wide consensus exists today in social science research on the need to complement income indicators, such as GDP, with additional social and environmental dimensions that complete the assessment of social progress (Costanza et al. 2009; Fitoussi and Stiglitz 2011). This enhanced view of well-being goes far beyond wealth and material standards of living and constitutes the social indicators (objective) approach to the measurement of QoL.^2^ It includes a list of domains which are not traded in markets but make life worth living (a clean environment or social relations, for instance) and reflect the normative ideals of society (Diener and Suh 1997).

Interest in QoL research accelerated after the findings of Easterlin (1974), Andrews and Withey (1976) or Campbell et al. (1976), who showed that economic growth (i.e., GDP growth) was not necessarily accompanied by the corresponding growth in well-being (the well-known Easterlin paradox).^3^ In the 1990s, the United Nations developed the Human Development Index, which complemented GDP with measures of health and education, with the aim of tracking social progress in developing and underdeveloped countries. The academic interest in the topic increased rapidly during the 1990s and 2000s. Institutions such as the OECD and the European Commission also showed strong interest in developing statistical tools for the assessment of the QoL in their respective domains. The influential report of the Commission on the Measurement of Economic Performance and Social Progress (CMEPSP) placed the topic at the centre of the social sciences agenda (Stiglitz et al. 2010).

Applied research in QoL has placed countries and individuals as the preferred units of analysis.^4^ In contrast, the municipal level has received much less attention. Data limitations partly explain this situation, since the indicators required to measure the various domains of QoL are only available for the largest cities. This is unfortunate, since the municipal level can be more relevant for the assessment of QoL than the regional or national levels. In a previous study (González et al. 2011), we found considerable differences in QoL across Spanish municipalities in 2001, even within the same province or region. Indeed, the municipal level explained the largest part of variation in well-being.

According to economic indicators, the recent financial crisis that started in 2008 had a profound impact on Europe and, in particular, on the Spanish population (Guardiola et al. 2015; Méndez et al. 2015). With negative growth of GDP (from 2009 to 2013) and alarming unemployment figures (peaking at 27 % as of January 2013), the risk of poverty and social exclusion has increased dramatically. It is estimated that 20 % of the Spanish population was below the poverty line in 2013, five points more than in 2004.^5^ The severe material deprivation rate also rose from 4.8 in 2004 to 6.2 in 2013.^6^ Our intention in this paper is to revisit the situation of the largest Spanish municipalities in terms of QoL in 2011 (i.e., 10 years after our previous study). For this purpose, we have carefully collected a comprehensive set of social and economic indicators covering all the relevant dimensions of QoL in 2011. This update may capture the impact of the crisis across the territory, not only in terms of GDP but also in terms of well-being.

Measuring QoL in municipalities is a demanding task. The dimensions to be accounted for are many and varied, but data at the municipal level are scant. While it is almost impossible to collect precise indicators to measure every single angle of QoL, in this paper we propose using various proxies that can be obtained from different sources. The basic data source for our research is the census microdata. We complement this source with mortality microdata, pollution records, official crime records and municipal financial reports. By combining different sources of information, we are able to provide a complete set of indicators related to every relevant dimension of QoL.

In order to aggregate this information into a composite index of QoL, we rely on data envelopment analysis (DEA).^7^ DEA is a frontier technique that has been extensively used for the measurement of efficiency in production. The method generates weights to aggregate all the dimensions considered in production (inputs and outputs) into a single index. While DEA was not initially designed for the measurement of QoL, its use within the social indicators literature has become increasingly popular, giving rise to the benefit of the doubt (BoD) approach (Cherchye et al. 2007). After the pioneering work of Hashimoto and Ishikawa (1993), who applied DEA to estimate QoL in Japan, more than 50 papers have applied this methodology for the measurement of QoL. See Mariano et al. (2015) for a comprehensive review.

In the estimation of the composite indicators of QoL, DEA generates the weights of the indicators in a manner that produces the most favourable evaluation possible for each municipality (BoD). However, this benevolence can also be seen as an important limitation of the technique, since the weights may be extremely different across municipalities and some dimensions that are considered essential in any definition of QoL (education, health, safety, etc.) may receive weights equal to 0 (therefore being implicitly considered unimportant). The use of weight restrictions can help in limiting these problems, while retaining some of the flexibility of DEA. In this research, to the traditional DEA specification, we will add weight restrictions to ensure a minimum reasonable representation of each QoL dimension in the final composite indicator. The robustness of this approach will be tested by altering the weight limits introduced and comparing the results. This method will be applied to compute QoL scores for the 393 Spanish municipalities with a population over 20,000.

The paper is structured as follows. Section 2 briefly reviews the literature on the measurement of the QoL and, in particular, its application to municipalities. Section 3 presents the data and describes the indicators used to approximate each of the eight dimensions of QoL considered. Section 4 describes the weighted constrained DEA model proposed. Section 5 presents and discusses the results, and concluding remarks are provided in a final section.

## The Measurement of Quality of Life in Municipalities

Social welfare is a central topic in Economics and other social sciences. Unfortunately, aggregate market-based indicators (GDP most notably), and not well-being measures, have traditionally guided policy decision making. The flaws of GDP are well known to economists (see Stiglitz et al. 2010) and there is growing consensus that the excessive political emphasis on aggregate market transactions is misplaced. Human and not economic development should be the ultimate goal of society. Furthermore, human development has a positive impact on economic growth, while the opposite is not necessarily true (Ranis and Stewart 2000). QoL measures can offer better guidance to policy making, since they summarize information about the many different dimensions of life that contribute to human development, welfare and, at the same time, sustainable growth.

During the last decade, the European Commission and the OECD have promoted interesting initiatives to introduce QoL into the political agenda, starting with the 2007 conference “Beyond GDP” followed by the 2009 conference “GDP and Beyond”, which challenged authorities and institutions to extend the focus of statistical information and political action beyond macroeconomic figures. The influential report of the French CMEPSP, elaborated by Stiglitz et al. in 2009, highlighted the multidimensional nature of QoL and sustainability, and specified the type of statistical information that should be developed to obtain useful indicators. Several institutions took up the challenge of developing such indicators, most notably the OECD and the European Statistical System (ESS). Since 2013, the OECD has published the Better Life Index and How is Life, addressing QoL through 11 dimensions (housing, income, jobs, community, education, environment, civic engagement, health, life satisfaction, safety and work-life balance). In turn, closely following the CMEPSP recommendations, the ESS Sponsorship group on Measuring Progress, Well-being and Sustainable Development, recommended 8 + 1 dimensions along which QoL should be addressed (material living conditions, productive or main activity, health, education, leisure and social interaction, economic and physical safety, governance and basic rights, natural and living environment, and overall experience of life).

While these efforts seem promising, the development of statistical information is still scant at the municipal level of analysis. Not surprisingly, most studies focus on the national or regional levels. Local information about the different dimensions of QoL is difficult to find for most cities within Europe. A notable contribution to extend the assessment of QoL to the local level is the Urban Audit Project (UAP), which started in 1999. The UAP compiles data in nine dimensions (demography, social aspects, economic aspects, civic involvement, training and education, environment, transport and travel, culture and leisure, and innovation and technology) with more than 300 variables corresponding to 284 European cities. It is a very ambitious project and has compiled a comprehensive collection of data which are very useful to construct rich composite indices of QoL. Unfortunately, the scope of the project is not yet large enough to allow the analysis of QoL at the municipal level within a given European country, since only the largest cities are included in the database (the type of information requested is only available for such large cities).

Despite data limitations there is a growing body of empirical literature estimating QoL in cities (Ballas 2013). Some early international examples include estimations of QoL for US metropolitan areas (Becker et al. 1989), Japanese prefectures (Hashimoto and Ishikawa 1993) or US counties (Marshall and Shortle 2005). Within Europe, Morais and Camanho (2011) used the Urban Audit data to compute composite QoL indicators for an extensive sample of 206 cities belonging to 25 countries. Within-country analyses in Europe are still scant—Poldaru and Roots (2014) is a recent example. In the case of Spain, the most comprehensive analysis measured QoL in a sample of 643 municipalities for 2001 (González et al. 2011). Other authors have estimated QoL indices for smaller intraregional samples, including Martin and Mendoza (2013) for the Canary Islands, Royuela et al. (2003) for the province of Barcelona, Zarzosa (2005) for the province of Valladolid or López and Sánchez (2009) for Galicia.^8^ Some recent research has estimated QoL indirectly by analyzing migration patterns in a sample of 700 Spanish municipalities (Navarro and Artal 2015).

The construction of a composite indicator of QoL at the municipal level is a challenging task, since QoL is a multidimensional construct that demands the availability of information about dimensions which are not readily available at that level of analysis. Data availability is thus a major limitation. In this paper, we try to overcome this limitation by making a considerable effort to collect data from sources containing information capable of approximating most of the dimensions of QoL identified in the literature. This has been a traditionally controversial issue. Diener (1995) proposed a QoL index based on the universal structure of values proposed by Schwartz (1992), which includes etic values that are recognized across cultures (e.g., enjoying life, protecting the environment, family security). Recent proposals, such as the influential Stiglitz et al. (2010) report, the subsequent work of the ESS Sponsorship group and the OECD’s “Better life” initiative, are also based on the recognition of these shared values. Following these sources, we propose an integrative framework that considers eight dimensions,^9^ for which information at the municipal level in Spain can be obtained. Table 1 shows the relationship between our proposal and the three sources just mentioned:

**Table 1:** Eight dimensions of QoL

	Our proposal	Stiglitz et al. (2010)	Sponsorship group	OECD
1	Material living conditions	Economic insecurity	Material living conditions	Income, housing
2	Health	Health	Health	Health
3	Education	Education	Education	Education
4	Environment	Environmental conditions	Natural and living environment	Environment
5	Economic and physical safety	Personal insecurity	Economic and physical safety	Safety, jobs
6	Governance and political voice	Political voice and governance	Governance and basic rights	Civic engagement
7	Social interaction	Social connections	Leisure and social interaction	Community
8	Personal activities	Personal activities	Productive and valued activities	Work-life balance

While there is no precise one-to-one link among the three specifications of QoL considered, they all focus on the same underlying factors. Of the eight dimensions, four are very precise and almost identically specified in the three proposals: health, education, environment, safety. We take sides with the ESS sponsorship group in combining economic and physical safety within the same dimension. The material living conditions dimension accounts for the income and housing dimensions suggested by the OECD’s Better Life Index, since they all refer to material conditions. In turn, personal activities accounts for activities other than work and is related to the OECD’s work-life balance. In the same manner, social interaction accounts for the concern for and connection with the community, which has been identified as a critical component of QoL. Finally, governance and political voice account for the participation of people in the political life of the municipality and the quality of public governance. In the following section we describe the data and indicators used to account for each of the eight dimensions of QoL considered.

## Indicators of QoL

Our objective in this paper is to measure QoL in 2011 for the largest Spanish municipalities using available data sources, updating our results of 2001. Our previous paper (González et al. 2011) relied mainly on data obtained from the census for a sample of 643 municipalities with a population over 10,000.^10^ The census is elaborated every 10 years by the Spanish *Instituto Nacional de Estadística* and contains varied information about people and dwellings. Unfortunately, only the municipalities with a population over 20,000 are identified in the 2011 census microdata. This has limited the current study to a reduced sample of 393 municipalities. The advantage of reducing the sample size in this manner is that richer statistical information is available for this smaller sample than for the original one. The new information that was not available in our previous study refers to mortality rates, crime and pollution records, volunteering activities and governance. Next we describe the battery of indicators that will be used to account for each of the eight dimensions of QoL considered in Table 1.

### Material Living Conditions

The first dimension focuses on the material or economic aspects of well-being and is strongly related to poverty and social exclusion. While we do not have information on per capita income at the municipal level for the entire sample, the census microdata provides a good proxy that is called the Average Socioeconomic Condition (ASC). This variable measures (on a scale) the socioeconomic status of every individual registered. Its municipal average is a reasonable proxy of material living conditions. A second element related to this dimension is housing, which is also partially associated with health concerns. From the census microdata we computed the Average Net Surface (ANS) and the average living conditions of the dwellings (LCD).^11^ By multiplying both variables we computed a combined indicator of the overall Quality of the Dwellings (QD = ANS × LCD).

### Health

Health is perhaps the most straightforward addition to GDP that is needed for obtaining a measure of well-being that goes beyond material concerns. Not surprisingly, health and education were the two key dimensions originally added to GDP by the United Nations in the development of the Human Development Index. In the case of between country comparisons, the most widely used health metrics are life expectancy and infant mortality rates; however, these dimensions may not be relevant for within country comparisons. In the case of Spain, geographical differences in life expectancy are small and differences in infant mortality are negligible. Instead of using those variables, in this paper we worked with mortality microdata to construct two indicators that reflect health differences within the Spanish territory.^12^

The first is excess of mortality (EM) adjusted by age. To construct this indicator for each municipality, we divided the population into age groups of 5 years (0–5, 6–10….) and then computed mortality rates within each age group. These rates were adjusted by weighting each age group rate by the national norm. This avoids attributing a higher mortality to municipalities in which the population is simply older. In other words, the index only varies if mortality rates within the different age groups vary across municipalities. The age-adjusted mortality rate of the municipality was then divided by the aggregate national mortality rate. This ratio reflects whether age-adjusted mortality in the municipality is higher or lower than the national norm. Then, we constructed a second indicator called avoidable mortality (AM). We counted the number of deaths that can be classified as avoidable following a consensus of Spanish health experts (Gispert et al. 2006). These include, for instance, breast cancer for females (not for males) in ages between 0 and 75. Health services should actively monitor females for breast cancer and available health services technology should be able to prevent this cause of death for patients younger than 75. Our AM variable is the ratio of avoidable deaths to total population in the municipality.^13^

### Education

The third component of the Human Development Index and a key dimension for a composite indicator of QoL is Education. The level of education increases subjective QoL (Ross and Van Willigen 1997) and additionally generates positive externalities on the community (Grace 1989). Therefore, it is not only the own education level that influences QoL but the joint education level of the community. The census microdata contain two relevant indicators of educational attainment. The first, and most informative one, is the overall level of education (OLE), on a scale from 0 (illiterate) to 10 (PhD). The census also provides a dummy variable indicating whether the individual completed a university degree (UD) or not.

### Environment

The physical urban environment plays a central role in limiting the potential for the development of good QoL and is also strongly related to sustainability. Environmental quality refers mainly to aspects such as the existence of clean green areas and unpolluted air and water, apart from other aspects more difficult to quantify such as the visual perception of the environment. Since 2007, the Spanish Ministry of Agriculture, Food and Environment has published data on the quality of air, obtained from a network of stations for measuring air quality. Most of the municipalities in the sample have one or several stations. In the cases of the few municipalities that did not have a station measuring air quality in 2011, we used the data of the nearest station (in all cases within a few kilometres). In the case of municipalities with more than one station (the large municipalities) we took the most negative data (i.e., maximum levels of pollution registered). The underlying assumption for this is that the population is also exposed to the worst conditions in the municipality. We compiled data on two different pollutants which are the subject of great concern for health according to the World Health Organization (WHO 2006): (1) Particulate matter (PM_10_, average daily value), which, according to the WHO, affects more people than any other pollutant. It is composed of small particles which can penetrate and lodge deep inside the lungs, contributing to many health problems such as lung cancer; and (2) Ozone (O_3_, 26th maximum 8-hour mean), which is one of the main components of photochemical smog and is associated with varied health problems, such as heart and lung diseases.^14^

### Economic and Physical Safety

Both economic and physical safety have been stressed as relevant components of the QoL. A usual indicator of economic safety is the Unemployment Rate (UR), a well-recognized source of economic insecurity and social exclusion. Further, unemployment is associated with a deterioration of physical and mental health (Lahelma 1992; Janlert 1997) and psychological well-being (McKee-Ryan et al. 2005). People who become unemployed report lower subjective QoL even after controlling for the loss of income (Fitoussi and Stiglitz 2011). Physical safety is also important, not only because of its most obvious effect on physical integrity, but also because of the effect of perceived insecurity on emotions (Stiglitz et al. 2010). Upon request, the Spanish Ministry of Home Affairs provided disaggregated crime data for all the municipalities in the sample, except those in País Vasco and Cataluña. Unfortunately, for these two regions we only had access to aggregate data.^15^ For this reason, we use the total number of crimes divided by total population (CRI).

### Governance and Political Voice

The quality of local governance greatly affects the quality of the public services received by citizens and, therefore, is of paramount importance to QoL. The financial condition of the local government can be used as a proxy of the quality of public management (e.g., Groves et al. 1981; Zafra-Gómez et al. 2009; Cuadrado-Ballesteros et al. 2012). Along this line, the financial result or cash surplus is a key indicator of financial health. In order to avoid the size effect of this indicator, we take the ratio of the cash surplus to the total budget of the local government (CS). In the same way, active participation of citizens in public decision making is a sign of freedom and concern about QoL. Political voice is critical for public policy accountability. The only available indicator of political voice for the whole sample of municipalities was the percentage of participation in municipal elections in 2011 (PME). Voter turnout is a common indicator for this dimension and has been used, for instance, in the OECD’s better life index within the civic engagement and governance domain.

### Social Interaction

The existence of places and institutions that facilitate social interaction can be beneficial to QoL since they ease developing social and cultural relations (Lloyd and Auld 2002). Involvement towards the community is also an important part of social interaction that contributes to QoL. Two indicators are available to be used as proxies for this dimension. The first, included in the census microdata, is participation in volunteering activities (VA), which shows the degree of commitment to the most needy in the community. The second variable is the total number of cultural and social centres available in the municipality, divided by the population (CSC).^16^

### Personal Activities

Related to the previous dimension is the time devoted to non-working pleasant activities. This is a very difficult dimension to measure with objective data, since it would also require subjective information about the satisfaction with those activities. Our municipal database contains two variables that reasonably approximate this dimension of QoL. The first is the commercial market share (CMS), a variable included in the Anuario Económico de España 2011 which is elaborated by La Caixa.^17^ This variable was already used by González et al. (2011) and indicates the proportion of commercial activity that takes place within the municipality boundaries in relation to the total commercial activity of Spain. As many of the pleasant personal activities identified by Stiglitz et al. (2010) imply consumption of some type, they will also contribute to the commercial market share of the municipality (e.g., shopping, travelling, eating, exercising). The second proxy is commuting time (CT), which negatively affects QoL since it takes away time from pleasant personal activities.^18^ Commuting has been consistently associated with reduced SWB even after compensating for the increased income or better housing that can be obtained from the extra income associated with larger commuting times (Stutzer and Frey 2008).

Table 2 shows the complete list of 16 indicators used to approximate the eight dimensions of QoL considered.^19^ It must be noted that some of these indicators contain information that overlaps across QoL dimensions. For instance, the variable CMS is included as a proxy for Personal Activities, but it can also be associated with Material Living Conditions, since the two dimensions overlap. Fortunately, the DEA model does not require matching each indicator with one or other dimension of QoL. In contrast, all 16 indicators will be entered independently in the estimation of the composite indicator, regardless of which dimension(s) they are related to.

**Table 2:** Partial indicators of the QoL dimensions

QoL dimension	Indicators
Material living conditions	Average socio-economic condition (ASC)
	Quality of dwellings (QD)
Health	Excess mortality (EM)I
	Avoidable mortality (AM)
Education	Overall level of education (OLE)
	Population with a university degree (UD)
Environment	Particulate matter (PM_10_)
	Ozone (O_3_)
Economic and physical safety	Unemployment rate (UR)
	Crime rate (CRI)
Governance and political voice	Local government cash surplus (CS)
	Participation in municipal elections (PME)
Social interaction	Population participating in volunteering activities (VA)
	Cultural and social centres (CSC)
Personal activities	Commercial market share (CMS)
	Commuting time (CT)

In sum, we have tried to overcome the traditional restrictions in data availability at the municipal level by compiling information from varied sources. Some treatment of the raw data was required in order to construct 16 indicators which, collectively, provide a fairly reasonable approximation to objective QoL conditions in the largest Spanish municipalities.

## Methods

As explained in the previous section, the first step to estimate the composite indicator of QoL was to compute the 16 indicators listed in Table 2 for each of the 393 municipalities in the sample. Then, these 16 indicators need to be aggregated into a single composite indicator. The OECD’s Handbook on Constructing Composite Indicators (Nardo et al. 2005) describes different methodologies that can be applied to combine varied information into a QoL index and the difficulties associated with each part of the process. Saisana et al. (2005) describe seven steps in which uncertainties arise in the construction of a composite indicator: selection of sub-indicators, data selection, data editing, data normalization, weighting scheme, weight’s values and composite indicator formula. Of these, “The most debated problem in building composite indicators is the difficulty in assessing properly the plurality of perspectives about the relative importance of the subindicators” (Saisana et al. 2005: 309). Ideally, weights should reflect the different importance that individuals attach to each of the underlying dimensions of QoL; however, importance varies from one individual to the next and it is controversial to determine empirically an appropriate set of weights.

Some proposals are based on revealed preference (Benjamin et al. 2014). Others, such as the OECD’s Better Life Index, ask individuals directly to rate the different dimensions of life. By the summer of 2015, a total of 2795 individuals from Spain had pointed to Health as their main concern, followed by Education and Work-Life Balance. Notably, on average, there are no great differences in the relative importance of the 11 dimensions of the Better Life Index; however, there is considerable variance across countries (also across gender and age). Therefore, we can also expect variation across municipalities within a given country. Equal weighting has also been extensively used in the literature. Equal weights have a very desirable property, since it has been shown that they can maximize consensus when individual preferences are heterogeneous (Hagerty and Land 2007). However, equal weighting is itself a type of value judgement.

The recognition of these difficulties for appropriate weighting of the sub-indicators called for methods that were data-driven. The conservative approach known as the benefit of the doubt (BoD) was first proposed by Melyn and Moesen (1991). The basic idea is to find the weights that maximize the composite indicator for the unit under analysis (in our case the municipal QoL score). This amounts to the assumption that any possible set of weights may be equally reasonable and, therefore (following this very conservative perspective), the researcher should select the one that gives the best possible evaluation of the municipality. DEA, a well-known non-parametric technique developed by Charnes et al. (1978) for measuring efficiency in production, does exactly this type of favourable weighting. Its application for the measurement of QoL was first proposed by Hashimoto and Ishikawa (1993) and has been used profusely since (Mariano et al. 2015).

Unfortunately, the extreme weight flexibility (benevolence) of DEA makes it highly sensitive to the presence of outliers, understood as municipalities with abnormally large values in some indicators. These municipalities will be placed on the DEA frontier even if the values of the other indicators are very low (Sharpe and Andrews 2012). In other words, “The optimization process can lead to many zero weights if no restrictions on the weights are imposed, so setting restrictions on weights is necessary for this method to be of practical use” (Vidoli and Mazziotta 2011: 265). Introducing weight restrictions can balance the need for weight flexibility (data-driven benevolence) with a reasonable degree of consistency. In any case, some weight restriction methods are more demanding than others in terms of value judgement. Interestingly, Mazziotta and Vidoli (2009) proposed a method in which weight restrictions are also data-driven, limiting external value judgement to the appropriateness of relating the weight of each sub-indicator to its own sample variance.^20^ In this paper we also aim at imposing minimum external value judgement in the weight restrictions introduced into the DEA programmes. We will also perform sensitivity analysis in order to check the robustness of the method proposed.

In order to compute the DEA scores, the first step is to construct a frontier containing the municipalities that must be considered as the best referents, assuming total flexibility on the weights of the different indicators of QoL. Let us follow the traditional specification of Charnes et al. (1978) with an output orientation, which requires solving the next mathematical programme for each municipality *i* in the sample:

1 $$\begin{aligned} \hbox{min} \;\frac{{\sum\nolimits_{m = 1}^{M} {v_{m} x_{im} } }}{{\sum\nolimits_{s = 1}^{S} {u_{s} y_{is} } }} \hfill \\ s.a:\hfill \\ \frac{{\sum\nolimits_{m = 1}^{M} {v_{m} x_{jm} } }}{{\sum\nolimits_{s = 1}^{S} {u_{s} y_{js} } }} \ge 1,\quad \forall j \hfill \\ u_{s} ,v_{m} \ge 0,\quad \forall s,m \hfill \\ \end{aligned}$$

where *x*_*im*_ represents the amount of input *m* in municipality *i*, *y*_*is*_ represents the amount of output *s* in municipality *i*, *v*_*m*_ is the weight of input *m,* *u*_*s*_ is the weight of output *s* and *j* represents any municipality in the sample.

González et al. (2011) used this model to compute QoL in Spanish municipalities, by defining a set of indicators as inputs (disadvantages of a city) and another set as outputs (advantages of a city). That approach seems very straightforward, since it fits directly with the traditional production setting of DEA.^21^ However, it is nonetheless problematic, since the DEA results may not be neutral to the selection of one indicator as an input or output. To avoid the arbitrary definition of the different indicators as either inputs or outputs (bads and goods), in this paper we preferred to transform all the variables into outputs (i.e., more is better) by applying a ratio-scale transformation. Conventional DEA models are units invariant and, therefore, a ratio-scale normalization of the data is acceptable (since it has no effect on the final results). We followed the “distance to the group leader” normalization method proposed by Cherchye et al. (2004). In the case of goods, we divided the value of the variable by its maximum (ASC, QD, OLE, UD, CS, PME, VA, CSC and CMS). In the case of bads, we divided the minimum of the variable by its value (EM, AM, PM_10_, O_3_, UR, CRI and CT). All the transformed variables vary from 0 to 1 and higher values indicate better QoL. After these transformations, we can compute a DEA composite indicator in which all the indicators are ouptuts (more is better) and we include an additional fictitious input variable which takes the value 1 for all municipalities. The resulting DEA model is equivalent to the estimation of the following composite indicator (Cherchye et al. 2007):

$$\begin{aligned} \hbox{max} \;\sum\limits_{s = 1}^{S} {u_{s} y_{is} } \hfill \\ s.t.:\hfill \\ \sum\limits_{s = 1}^{S} {u_{s} y_{js} } \le 1,\quad \forall j \hfill \\ u_{s} \ge 0,\quad \forall s \hfill \\ \end{aligned}$$

This programme finds the weights *u*_*s*_ that maximize the composite indicator for municipality *i*. The constraint imposes a frontier on the sample by forcing the value of the composite indicator of all the municipalities to be less than some fixed value (typically 1) which establishes the frontier. If municipality *i* is on the QoL frontier, then the objective function will reach the value 1, since no other municipality will be able to obtain a higher weighted sum, with the most favourable set of weights for municipality *i*. In contrast, underperformers can only attain values lower than 1 for the objective function. In this case, even with their best possible set of weights, there exists another municipality which obtains a higher weighted sum. Therefore, the score will be bounded within the (0,1] interval, with values lower than 1 reflecting the distance to the QoL frontier.

As we mentioned earlier, a distinctive feature of DEA is the absolute flexibility in the way the linear programme can apply any possible set of weights for each municipality in the sample. Recall that the programme is solved independently for each municipality and, therefore, optimal weights may be completely different from one municipality to another. The main argument favouring this extreme weight flexibility is that, given our ignorance on the appropriate weight structure, this procedure will make an evaluation of the municipality under its most favourable scenario (BoD). The idea is that the observation that an indicator has a larger value in a municipality may reflect the greater importance of that dimension for the population of that municipality. The DEA index is conservative enough to allow for this possibility.

On the other hand, complete weight flexibility does not seem reasonable. In practice, we end up with completely different data-driven sets of weights across municipalities. And these sets often include weights equal to zero (to neutralize indicators in which the municipality has a low value). Is it reasonable to assume that the citizens of municipality A do not care at all about crime (just because they suffer from high crime rates and they score low in that indicator), while the citizens of municipality B have the highest concern about crime (simply because they have comparatively low crime rates)? If we accepted that, we would need to revise the definition of QoL itself and conclude that QoL is something completely different for municipalities A and B. It simply does not seem realistic. Unconstrained DEA may (and will) produce such absurd results in empirical applications. It is common to have a large number of indicators receiving zero weights, simply because the values of those variables are not large enough to deserve a positive weight. To maximize the QoL index, the DEA programme assigns positive weights only in the most favourable indicators. This is a well-known flaw in the DEA literature and many different solutions have been suggested, which imply restricting the range of acceptable values for the weights (Thompson et al. 1986; Dyson and Thanassoulis 1988; Allen et al. 1997; Roll et al. 1991; Wong and Beasley 1990; Pedraja et al. 1997; Sarrico and Dyson 2004). In the words of Vidoli and Mazziotta (2011: p.265) “setting restrictions on weights is necessary for this method to be of practical use”.

A controversial issue in weight restrictions literature is the establishment of an acceptable range of weights. In terms of value judgement, some methods are more demanding than others. In our previous research (González et al. 2011) we tried to minimize the degree of external value judgement following an indirect approach known as value efficiency analysis (VEA) (Halme et al. 1999). VEA restricts the set of acceptable weights to those which bring a pre-selected municipality (the Most Preferred Solution, MPS) to the frontier. By selecting an exemplar municipality (the MPS) the VEA programme internally finds the weights which are most favourable to each municipality under analysis but such weights must be reasonable for the MPS at the same time. The major problem with this approach is, obviously, the selection of the MPS (Korhonen et al. 1998). A second issue with VEA is that it does not guarantee that some indicators receive zero weights. Zero weights in some indicators (it’s less favourable) can be reasonable for any MPS selected.

Instead of using value judgement to select an ideal MPS, in this paper we propose a classic weight restrictions scheme, which combines a degree of flexibility with an equivalent degree of weight consistency. The basic idea comes from comparing the two extreme solutions of unconstrained DEA and equal weighting with an intermediate compromise solution in which 50 % common weight is imposed, while 50 % flexibility is allowed. Therefore, we propose a balanced trade off by imposing the constraint that each of the 16 partial indicators must have at least one half of the weight share it would have under an equal weighting scheme and no more than one half more. In other words, at least half of the weighting must be common for all the municipalities in the sample (16 × 3.125 % = 50 %) while the other half will be discretional for each municipality, with 50 % discretionality within each indicator. In this manner, we follow Wong and Beasley (1990) in order to restrict the shares of each of the 16 indicators in the following manner:

$$0.03125 \le \frac{{u_{k} y_{k} }}{{\sum\nolimits_{s = 1}^{16} {u_{s} y_{s} } }} \le 0.09375,\quad k = 1 \ldots 16$$

A good property of this approach to weight restrictions is that the resulting composite indicator still remains invariant to the units of measurement (Cherchye et al. 2007: 132). The process is able to combine a degree of weight flexibility with the same degree of consistency in weighting. With 16 indicators, the 50 % common weighting, translates into a 3.125 % minimum weight for each indicator and a maximum of 9.375 %. Under equal weighting, all the indicators would receive an equal weight of 6.25 %. A variation of 50 % either up or down is allowed in our proposal. Indicators with a low value may receive the lowest weight of 3.125 % (50 % lower than the corresponding equal weight). Conversely, indicators in which the municipality performs well may receive weights as large as 9.375 % (50 % larger than the corresponding equal weight). In any case, the particular weighting vector of each municipality will be data-driven (within these limits), being the most favourable to each municipality. The resulting weights will therefore be halfway between equal weighting and unrestricted BoD weighting.

In order to check for the robustness of this scheme of weight restrictions, we must compare the results obtained under several different weighting schemes. Unconstrained DEA represents an extreme solution in which each indicator’s share ranges from 0 to 1. In contrast, equal weighting represents the other extreme solution in which each indicator’s share is exactly 1/16. Our proposed solution is located halfway, with a 50 % common weighting scheme. However we may also compute the QoL scores under other intermediate schemes. We will try with 30, 40, 60 and 70 % common weight as shown in Table 3.

**Table 3:** Alternative weighting schemes to check for robustness

Weighting scheme	Lower bound	Upper bound
Unconstrained DEA	0	1
30 % common	0.01875	0.10625
40 % common	0.025	0.1
50 % common	0.03125	0.09375
60 % common	0.0375	0.0875
70 % common	0.04375	0.08125
Equal weighting	0.0625	0.0625

Our expectation is a high degree of correlation between all the intermediate options suggested. In contrast, correlation with unconstrained DEA may be weak, since this method can lead to very extreme weights as discussed above. Unconstrained DEA will bring too many municipalities to the QoL frontier (or close to), simply because they excel in one single indicator. These solutions are ruled out under any of the weight restricted specifications.

## Results

As discussed earlier, our analysis is based on 16 indicators that cover the eight dimensions of QoL considered (two indicators per dimension). Before presenting the results, we will briefly describe the geographical differences that can be directly appreciated from the description of these eight dimensions. In order to simplify the presentation of this information, we aggregated the municipal data at the level of the Autonomous Community (AC).^22^ Table 4 shows descriptive statistics for the eight dimensions of QoL.^23^ The first column shows the number of municipalities in the sample belonging to the AC and the percentage of the population of that AC which is represented in the sample (in brackets). On average the 393 municipalities of our sample cover 68 % of the Spanish population, even though they only represent 5 % of the 8122 municipalities in Spain.^24^ Although our sample offers a fairly good representation of the Spanish population, some ACs are better represented than others, because they concentrate larger fractions of the population in densely populated areas. Madrid is the best represented AC in our sample, with more than 90 % of the population, followed by Murcia (82.5 %), Canarias (76.8 %) and Comunidad Valenciana (72 %). In contrast, more rural ACs are not as well represented in the sample, especially Navarra (39.4 %), Extremadura (40.1 %) and Castilla-La Mancha (40.5 %). The coverage of remaining ACs varies between 50.8 % (Castilla y León) and 70.8 % (Baleares).

**Table 4:** Eight QoL dimensions by autonomous community

	N (coverage %)	Material living conditions	Health	Education	Environment	Economic and physical safety	Governance and political voice	Social interaction	Personal activities
Andalucía	81 (67.7)	0.631**↓**	0.373**↓**	0.544	0.564	0.282**↓**	0.654	0.457	0.681
Aragón	4 (58.5)	0.738	0.409	0.622	0.522	0.387	0.700	0.480	0.654
Asturias	7 (69.4)	0.695	0.363**↓**	0.588	0.539	0.438**↑**	0.720	0.396**↓**	0.660
Baleares	12 (70.8)	0.699	0.406	0.538	0.594**↑**	0.392	0.646	0.461	0.754**↑**
Canarias	25 (76.8)	0.588**↓**	0.396	0.517**↓**	0.472**↓**	0.294**↓**	0.710	0.426	0.680
Cantabria	5 (54.0)	0.728	0.407	0.597	0.590**↑**	0.403	0.740	0.376**↓**	0.706
Castilla y León	15 (50.8)	0.726	0.436	0.611	0.520	0.382	0.698	0.437	0.708
Castilla-La Mancha	15 (40.5)	0.730	0.447	0.572	0.528	0.345	0.727	0.442	0.702
Cataluña	63 (70.3)	0.712	0.418	0.580	0.562	0.333	0.637	0.479	0.628**↓**
Com. Valenciana	63 (72.0)	0.675	0.388	0.548	0.541	0.297**↓**	0.723	0.453	0.691
Extremadura	7 (40.1)	0.684	0.406	0.601	0.553	0.326	0.763	0.478	0.715
Galicia	22 (51.4)	0.722	0.412	0.591	0.520	0.388	0.717	0.415	0.686
Madrid	32 (90.3)	0.740	0.508**↑**	0.672**↑**	0.535	0.376	0.695	0.416	0.574**↓**
Murcia	17 (82.5)	0.679	0.397	0.520**↓**	0.517	0.378	0.708	0.436	0.710
Navarra	3 (39.4)	0.774**↑**	0.450**↑**	0.673**↑**	0.540	0.439**↑**	0.722	0.567**↑**	0.743
País Vasco	18 (64.4)	0.727	0.406	0.630	0.588**↑**	0.423**↑**	0.693	0.492	0.653
La Rioja	2 (55.2)	0.735	0.436	0.598	0.605**↑**	0.402	0.741	0.529**↑**	0.762**↑**
Ceuta/Melilla	2 (100.0)	0.536**↓**	0.346**↓**	0.513**↓**	0.530	0.229**↓**	0.653	0.473	0.787
Total Average	393 (68.0)	0.696	0.411	0.584	0.546	0.362	0.694	0.456	0.694
Total SD	393 (68.0)	0.059	0.036	0.048	0.034	0.058	0.071	0.046	0.051

The table shows the average values for each of the eight dimensions of QoL considered and, also, an arrow indicating whether the value is higher than the national average plus 1 standard deviation (**↑**) or lower than the national average less 1 standard deviation (**↓**). Navarra emerges clearly as the AC with the best overall profile, since it shows five upward arrows. It is closely followed by La Rioja and País Vasco. All these three ACs share the same geographical area in the central north part of Spain and obtained very high QoL scores in our previous study for 2001 (González et al. 2011). Conversely, the most negative profiles are observed in the autonomous cities of Ceuta/Melilla and Canarias (four downward arrows), and Andalucía (three downward arrows). This observation is also consistent with our previous results for 2001. In between, we observe ACs with intermediate profiles, i.e. not too high and not too low in the eight QoL dimensions (Aragón, Castilla y León, Extremadura and Galicia).

The previous description of the QoL dimensions provides a first approach to the geographical profiles of QoL; however, our ultimate objective is to condense all this information into a single composite indicator of QoL. Since we also want to check the robustness of the aggregation method, we have computed the QoL scores under the seven different specifications indicated in Table 3. The aggregate results by AC are shown in Table 5. The first column shows the (population weighted) average of the QoL score computed under the conventional unconstrained DEA specification (i.e. without any type of weight constraints). Conversely, the last column contains the averages under an equal weighting scheme. Between these extremes we show the results obtained when a given minimum common share is imposed on each indicator (ranging this minimum from 30 to 70 % of the corresponding equal weighting share).

**Table 5:** Summary of QoL scores by autonomous region under different weighting schemes

	Weight constrains						
	Unconstrained	30 %	40 %	50 %	60 %	70 %	Equal
Andalucía	0.915	0.800	0.776	0.750	0.725	0.700	0.632
Aragón	0.948	0.880	0.866	0.851	0.836	0.819	0.763
Asturias	0.941	0.865	0.847	0.824	0.796	0.767	0.691
Baleares	0.947	0.852	0.827	0.802	0.777	0.753	0.682
Canarias	0.949	0.823	0.796	0.776	0.740	0.712	0.636
Cantabria	0.972	0.886	0.864	0.843	0.819	0.793	0.721
Castilla y León	0.955	0.886	0.869	0.851	0.831	0.809	0.742
Castilla-La Mancha	0.966	0.865	0.846	0.827	0.808	0.786	0.722
Cataluña	0.957	0.825	0.802	0.777	0.749	0.721	0.643
Com. Valenciana	0.949	0.845	0.821	0.796	0.769	0.742	0.670
Extremadura	0.986	0.900	0.880	0.859	0.835	0.811	0.739
Galicia	0.958	0.886	0.867	0.845	0.822	0.796	0.725
Madrid	0.958	0.847	0.830	0.811	0.793	0.775	0.714
Murcia	0.931	0.842	0.822	0.801	0.777	0.754	0.688
Navarra	1.000	0.945	0.928	0.910	0.891	0.870	0.807
País Vasco	0.969	0.893	0.876	0.857	0.836	0.812	0.747
La Rioja	1.000	0.950	0.930	0.909	0.882	0.856	0.784
Ceuta/Melilla	0.959	0.788	0.762	0.735	0.707	0.682	0.614
Total	0.948	0.843	0.821	0.799	0.775	0.751	0.682

By construction, the DEA-QoL scores are smaller the more constrained the weights are. Therefore, the numbers are not directly comparable across columns. However, within each column, they are indicative of the relative differences in the QoL of the different ACs. Table 6 contains the QoL ranks under the seven specifications computed. The North and Central regions show the highest QoL under all the different specifications and, therefore, occupy the best positions in the rankings. Navarra, La Rioja, País Vasco and Extremadura appear consistently the first positions. Other Central and Northern regions, such as Cantabria, Castilla y León, Aragón or Galicia also achieve very positive results. In contrast, the Southern and Mediterranean regions (including the islands) obtain the lowest scores. Ceuta/Melilla, Andalucía, Canarias, Cataluña, Com. Valenciana, Murcia, Baleares are consistently found within the worst positions in the rankings.

**Table 6:** QoL ranking of autonomous regions under different weighting schemes

	Weight constraints						
	Unconstr.	30 %	40 %	50 %	60 %	70 %	Equal
Andalucía	18	17	17	17	17	17	17
Aragón	14	8	7	5	3	3	3
Asturias	16	9	9	10	10	11	11
Baleares	15	11	12	12	12	13	13
Canarias	13	16	16	16	16	16	16
Cantabria	4	6	8	8	8	8	9
Castilla y León	11	5	5	6	6	6	5
Castilla-La Mancha	6	10	10	9	9	9	8
Cataluña	10	15	15	15	15	15	15
Com. Valenciana	12	13	14	14	14	14	14
Extremadura	3	3	3	3	5	5	6
Galicia	8	7	6	7	7	7	7
Madrid	9	12	11	11	11	10	10
Murcia	17	14	13	13	13	12	12
Navarra	1	2	2	1	1	1	1
País Vasco	5	4	4	4	4	4	4
La Rioja	1	1	1	2	2	2	2
Ceuta/Melilla	7	18	18	18	18	18	18

Table 7 shows the Pearson correlation coefficients among the seven QoL composite indicators (figures above the main diagonal) and also the Spearman-rank correlations (figures below the main diagonal). The values for both correlation coefficients are very similar. Correlations are very high among six of the seven weighting schemes considered (most of them above 0.90). The only exception is the unconstrained conventional DEA scores, which correlate low with the equal weighting composite indicator (0.389) and attain their highest correlation with the 30 % share constrained indicator (0.662). This result points to the inconsistency (lack of robustness) of unconstrained DEA.

**Table 7:** Pearson (top-right) and spearman rank (bottom-left) correlation of QoL composite indicators

	Weight constraints						
	Unconstrained	30 %	40 %	50 %	60 %	70 %	Equal
Unconstrained	1	0.662	0.610	0.560	0.517	0.479	0.389
30 %	0.680	1	0.991	0.970	0.946	0.920	0.848
40 %	0.622	0.989	1	0.993	0.978	0.959	0.900
50 %	0.567	0.965	0.991	1	0.995	0.984	0.939
60 %	0.517	0.936	0.973	0.994	1	0.997	0.965
70 %	0.477	0.908	0.952	0.981	0.996	1	0.983
Equal	0.367	0.823	0.882	0.926	0.958	0.978	1

This idea is reinforced by the observation that unconstrained DEA QoL scores are well above 0.90 even in the worst performing regions. These very large values certify the failure of unconstrained DEA to provide a realistic composite indicator of the QoL. With no weight constraints it is easy to find a single dimension or a combination of a few dimensions in which most municipalities are comparatively good. The DEA programme simply assigns very large weights to these dimensions and zero weights to the rest. Our empirical estimation illustrates this problem well, since 63.9 % of the weights received a value of 0 in the computation of the unconstrained DEA composite indicator. There is even a weighting vector that takes Ceuta/Melilla close to the QoL frontier (to the 7th position in the ranking). Strikingly, Ceuta/Melilla have four of the eight dimensions of QoL below one standard deviation of the mean, as shown in Table 4. In this particular case, the DEA programme found a vector with zero weights for those five dimensions and positive weights for the other three, with a very high weight placed on the Personal Activities dimension, in which these two cities are well above the average.

As evidenced by our results, the extreme weight flexibility of unconstrained DEA leads to solutions with zero weights in the indicators in which municipalities perform poorly and high weights to the indicators in which they perform well. These results call for weight restrictions in order to achieve a reasonable degree of congruence in the construction of the composite indicator. Our results also show that it is not essential to ask for a very high degree of congruence in the weightings. Even with the slighter weight constraints imposed in our empirical exercise (30 % common weight) the results are very similar to those obtained with the most demanding weight constraint scheme (equal weighting, i.e. 100 % common weight). The correlation between these two composite indicators is 0.848 and this figure increases to 0.920 with the 70 % common weight scheme. The rankings are very similar for the great majority of the regions under any of the six weight restricted schemes. The only notable exception is Aragón, which ranks 3rd under equal weighting and 8th under 30 % common weight.

Therefore, our results suggest that, while it is important to constrain weights in the computation of composite indicators of QoL, the exact range of weights used is not equally important. Any type of weight restriction rules out the possibility of zero (or very small) weights for the indicators. And doing that makes sense, since all the indicators which are included in the analysis are so because they were considered relevant to measure some dimensions of QoL. The average weight shares of each of the 16 indicators used in the analysis can be seen in Table 8. With unconstrained DEA there is a wide disparity of shares, ranging from 1.3 % of UD (Education) to 21.1 % of PME (Governance and Political Voice). Indeed, half of the indicators have average shares below 3 %. As pointed out before, 63.9 % of the individual weighting shares are 0 under this scheme. It is also noticeable that for eight municipalities, 100 % of the share corresponds to one single indicator (having 0 % shares in the other 15). Weight restrictions correct these extreme solutions. Under the 50 % common weight scheme, weight shares are forced to vary within the range 3.125–9.375 %. As we can see, some indicators (QD, UD, UR) still receive average weights close to the minimum of the acceptable range. Alternatively, other indicators (O3, CMS, CT) get close to the maximum; however, the majority of them show average shares in the middle of the interval. By QoL dimension, Personal Activities, Governance and Political Voice and Environment have the largest weight shares, while Health, Education and Safety have the lowest. Similar results are obtained under the other weight constrained DEA specifications.

**Table 8:** Average weight shares (%) of the 16 indicators under the different weight schemes

	Weight constraints						
	Unconstrained	30 %	40 %	50 %	60 %	70 %	Equal
ASC	13.3	9.3	8.7	8.2	7.6	7.1	6.25
QD	2.3	2.7	3.1	3.6	4.1	4.7	6.25
EM	2.3	5.3	5.4	5.3	5.2	5.5	6.25
AM	1.9	3.4	3.7	4.0	4.4	4.9	6.25
OLE	4.1	6.6	5.9	5.5	5.3	5.3	6.25
UD	1.3	2.1	2.7	3.3	4.0	4.6	6.25
O_3_	11.5	10.1	9.5	9.1	8.5	7.9	6.25
PM_10_	2.9	5.0	5.5	6.0	6.3	6.5	6.25
CRI	2.0	4.6	5.3	6.0	6.3	6.5	6.25
UR	1.9	2.3	2.9	3.4	4.0	4.6	6.25
PME	21.1	9.3	8.7	8.1	7.7	7.3	6.25
CS	6.4	8.3	8.2	7.8	7.5	7.2	6.25
VA	5.3	5.0	4.6	4.5	4.7	5.1	6.25
CSC	2.0	5.4	6.3	6.9	7.3	7.2	6.25
CMS	11.1	10.3	9.8	9.2	8.5	7.9	6.25
CT	10.7	10.2	9.7	9.1	8.5	7.9	6.25

To sum up, Navarra and La Rioja lead the QoL frontier in any specification. País Vasco, Castilla-León, Aragón, Extremadura, Galicia and Cantabria are also close to the frontier in all the weight constrained specifications. In contrast, Canarias, Ceuta/Melilla, Andalucía, Cataluña, Com. Valenciana, Murcia, Baleares obtain the lowest QoL averages. Paradoxically, the municipalities with the highest QoL locate within the AC of Madrid, which has only a moderate average. If we focus on our proposal of setting 50 % common weight, the only municipality obtaining a QoL score equal to 1 (i.e., the maximum possible value) is Pozuelo de Alarcón, closely followed by Boadilla del Monte, Tres Cantos and Mahadahonda with 0.985. All these municipalities belong to the AC of Madrid. Behind them, there is a group of six very high QoL municipalities (with scores above 0.95) from the ACs of País Vasco, Madrid and Aragón: Teruel (0.982), Zarautz (0.974), Huesca (0.964), Leioa (0.956), Las Rozas de Madrid (0.953) and Arrasate/Mondragon (0.952). At the bottom we find municipalities from Canarias (Mogán, 0.417), Andalucía (La Línea de la Concepción 0.438; Cártama, 0.506) and Cataluña (Salt, 0.521).

Table 9 shows the top 10/bottom 10 QoL rankings of the 81 municipalities which are either provincial capitals, AC capitals or have a population over 100,000. The table shows the QoL score and the position of the municipality within the overall ranking of the 393 municipalities included in the sample. Teruel is the first provincial capital to appear in the ranking, while it occupies the fifth position in the overall ranking with a score of 0.982. In the top ten we find similar small and medium sized capitals from the central and northern regions of Spain. In this list, only San Sebastián, Pamplona and Logroño are over the 100,000 population. In contrast, in the bottom ten we find large and medium sized municipalities which are not provincial capitals (except Málaga and Huelva). They locate principally in the Southern regions (including Canarias), and the ACs of Cataluña and Madrid.

**Table 9:** QoL ranking of large municipalities and provincial capitals 2011

Municipality top 10	Rank	Score	Municipality bottom 10	Rank	Score
Teruel	5	0.982	Huelva	300	0.747
Huesca	7	0.964	Málaga	309	0.743
Santiago de Compostela	13	0.941	Jerez de la Frontera	326	0.735
Soria	16	0.925	Fuenlabrada	333	0.730
San Sebastián	17	0.922	Ceuta	344	0.720
Cuenca	18	0.921	Badalona	356	0.709
Toledo	19	0.919	Telde	361	0.704
Logroño	20	0.917	Santa Coloma de Gramenet	367	0.693
Pamplona	21	0.913	Algeciras	374	0.677
Cáceres	22	0.912	Parla	380	0.665

If we focus on the ten largest municipalities, Valencia and Zaragoza obtain the highest QoL with scores of 0.838 and 0.836, respectively. They are followed by Bilbao (0.832), Murcia (0.823) and Madrid (0.816). In contrast, Málaga (0.743), Sevilla (0.758) and Las Palmas de Gran Canaria (0.774) obtain the lowest scores.

**Fig. 1:**
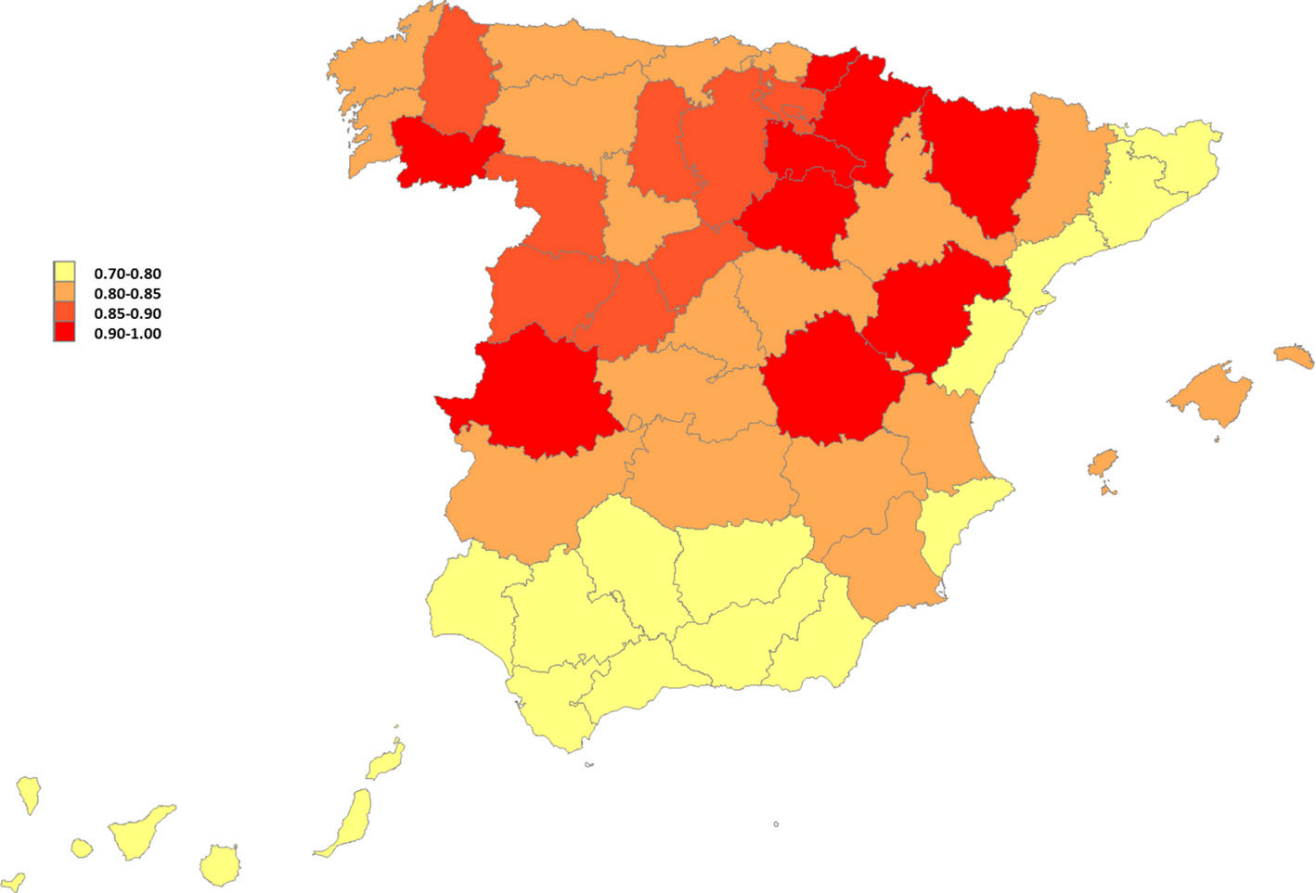
QoL in Spanish provinces

Figure 1 shows the geographical distribution of the QoL in the 52 Spanish provinces under the 50 % common weight scheme. The lowest levels of QoL (yellow areas) are displayed in Canarias, the provinces of Andalucía and the provinces on the Mediterranean coast except Valencia and Murcia (Alicante, Castellón, Tarragona, Barcelona and Gerona). The provinces in red achieve the largest QoL indices and are concentrated in the central north part of Spain (Guipuzcoa, Navarra, La Rioja, Soria, Huesca, Teruel and Cuenca) and the western provinces of Cáceres and Orense. In general terms, the Northwest obtains a good evaluation. This distribution is similar to the one depicted for 2001 by González et al. (2011). The most notable difference refers to the Mediterranean provinces of Cataluña and Valencia, which performed fairly well in 2001 and turned to yellow in 2011. The deterioration in the QoL of the Mediterranean regions has also been documented by Navarro and Artal (2015). The high degree of vulnerability of the Mediterranean regions to face the economic crisis, as reported by Méndez et al. (2015) for the period 2006–2012, explains this negative trend in that part of Spain.

**Fig. 2:**
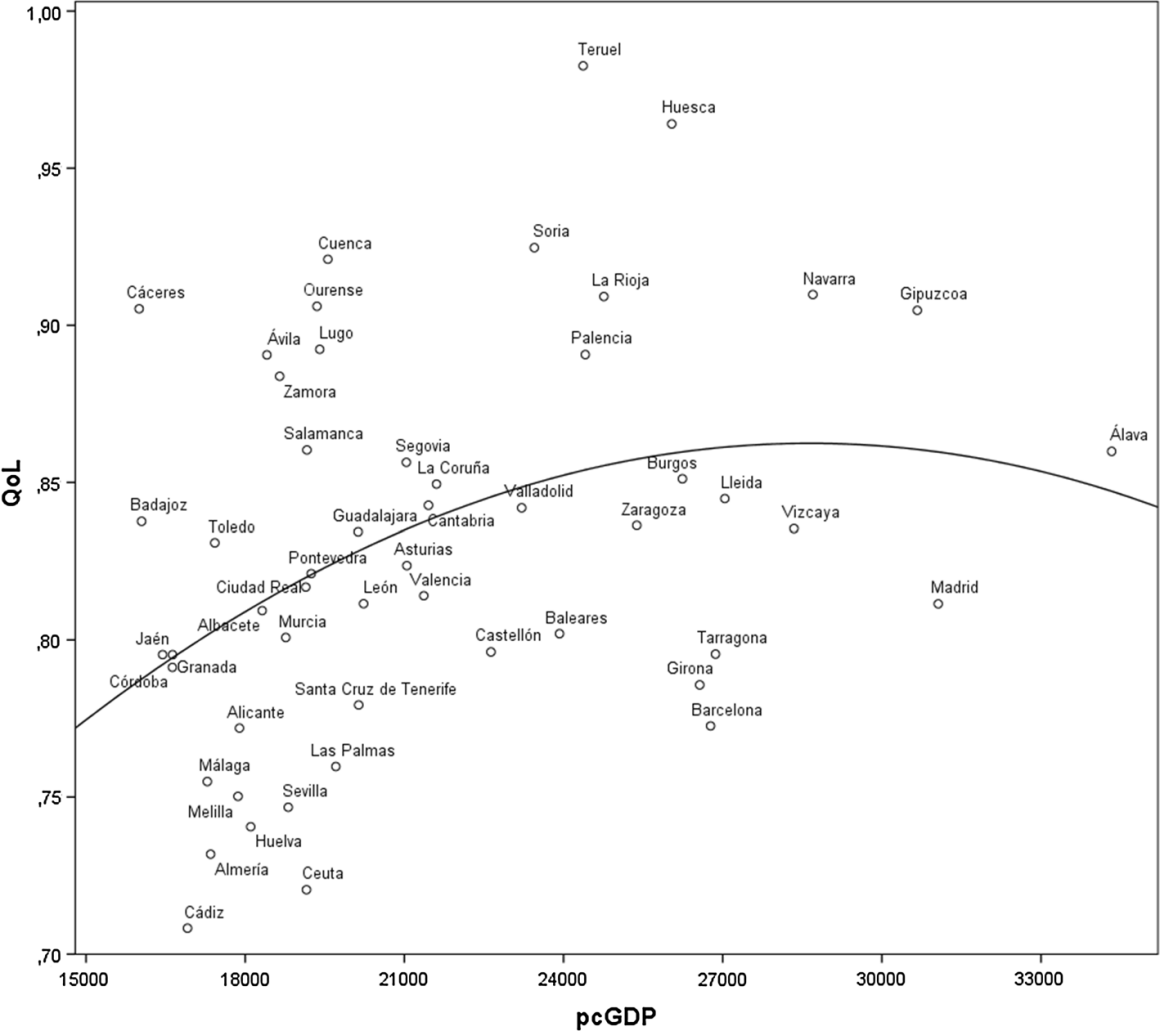
Relationship between provincial per capita GDP and QoL

As a final exercise, we explored the relationship between GDP and QoL. For this purpose, we used per capita GDP data at the provincial level. It would be desirable to use municipal data, but unfortunately income or GDP data are not available at that level of analysis.^25^ Figure 2 shows the relationship between our QoL index and per capita GDP in the 52 Spanish provinces. While we find a statistically significant inverted U-shape correlation between the two variables, the figure also shows large departures from the regression curve. Central-West and Central-North provinces, such as Teruel, Huesca or Cáceres, have QoL well above the line, while Southern and Mediterranean provinces are considerably below the line. The largest provinces, those with more than 1 million population (Madrid, Barcelona, Valencia, Alicante, Málaga, Sevilla, and Cádiz), are all below the line, reflecting the costs of large urban areas in terms of QoL. In contrast, the smallest provinces (Teruel, Soria, Huesca, Segovia, Cuenca, Ávila, Zamora, Palencia and Lugo) are mostly above the line.

## Concluding Remarks

Measuring QoL involves collecting information on a wide variety of indicators covering the different facets of well-being. Traditionally, economic indicators adjusted with health and education have been used extensively, inspired by the Human Development Index of the United Nations. Today, that approach seems clearly incomplete. Different experts and institutions recommend using an exhaustive list of indicators covering material aspects of well-being, health and education, but also the environment, safety, governance, social interaction and personal activities. The problem with this approach is that when the unit of analysis falls from nation to region and from region to municipality it becomes increasingly difficult to obtain the data which are needed in order to cover all the domains of QoL appropriately.

Throughout this research, we have systematically revised all the available data sources that contained potentially useful information related to eight dimensions proposed for measuring QoL at the municipal level in Spain. While some indicators could be easily obtained from conventional official statistics (unemployment or political voice, for instance), others were carefully obtained after soliciting microdata from different sources and after laborious statistical treatment of those data (avoidable mortality, excess mortality or quality of dwellings, for instance). The final outcome of this work was a complete set of 16 indicators, two per each of the eight dimensions of QoL considered, for all the Spanish municipalities with a population over 20,000. This sample represents 68 % of the entire Spanish population, although some unpopulated regions are underrepresented given their rural municipal structure (for example, Navarra or Extremadura). This unique data set allows making comparisons among municipalities and regions in terms of QoL.

Through a DEA programme, we combined the 16 variables into a composite indicator, following the normalization proposed by Cherchye et al. (2004). To avoid the well-known deficit in the discriminating power of DEA when there is complete weight flexibility, we imposed a structure of weight restrictions forcing 50 % of the weighting shares to be common across municipalities. We believe this structure allows for enough discretion (needed to account for differential priorities under a “benefit of the doubt” approach), while assuring at the same time a desirable degree of consistency in weighting. In order to check the robustness of this approach we also computed the results with unconstrained DEA, equal weighting and four different weight constraint schemes being more and less demanding than the 50 % common weight scenario proposed originally. The results show a very high correlation among all the QoL composite indicators computed under the six different weight restricted schemes. The only exception is unconstrained DEA, which produces 63.9 % zero weights and does not correlate highly with any of the other specifications. From this observation we conclude that, while it is important to introduce weight constraints in order to avoid the zero weighting problem, the actual weight constraints which are imposed are not of significant importance.

We find the Central-North regions comprising the highest QoL averages and the Southern and Mediterranean regions showing the lowest performance. This finding is consistent with the identification of the Mediterranean and Southern regions as the most exposed to the recent financial crisis. Future research should establish a direct comparison with the situation in 2001. This comparison will allow identifying catching-up movements and also shifts in the QoL frontier.

## Footnotes

1 We must note that GDP was not originally designed as a measure of social progress or well-being, but simply as a measure of economic activity. However, it has been and still is commonly used by both economists and politicians as a proxy of well-being.

2 In contrast, subjective well-being (SWB) research focuses on the individual’s internal judgement of his or her own life.

3 QoL research can be traced back much earlier (see Sirgy et al. (2006) for a comprehensive review).

4 See Reig-Martinez (2013) and Somarriba et al. (2015) for recent examples.

5 Data obtained online from the official statistics of the Instituto Nacional de Estadística.

6 Data obtained online from the official statistics of Eurostat.

7 There are numerous alternative approaches to the construction of composite indicators of QoL (see Nardo et al. 2005; Hagerty et al. 2001).

8 Other authors have focused on the study of QoL in the Spanish regions or provinces, for which statistical information is more developed (Murias et al. 2006; Jurado and Perez-Mayo 2012).

9 The ESS sponsorship group proposes a 9th dimension (Overall Experience of Life) to account for the subjective perception of their own QoL; so does the Better Life Index of the OECD. While we sympathize with the life satisfaction dimension, in this study we only have access to objective dimensions (not self-reported perceptions). Therefore, our QoL index will be constructed in the tradition of the Scandinavian welfare research (Erikson 1974, 1993) that bases QoL exclusively on objective indicators. It can also be understood in terms of the capabilities approach (Sen 1993).

10 This census is named “Censo de Población y Viviendas” and is elaborated by the Spanish National Statistics Office (INE) every 10 years. The last one referred to 2011.

11 This index ranges from 0 to 100 and takes into account factors of the buildings, such as the age since construction, tumbledown status, hygienic conditions, running water, indoor toilet, accessibility, heating, etc.

12 Mortality microdata include the complete registry of deaths, including the cause of death, age and residence. The microdata identifying the municipality of residence are not publicly available. We thank the INE for facilitating access to these data for our research.

13 To be exact, we used the data of the population under 75, since most cases of death are only considered to be avoidable for individuals below that cut-off age.

14 The WHO also stresses the importance of nitrogen dioxide (NO_2_) and sulfur dioxide (SO_2_). Unfortunately, data for these two elements were not available for the entire sample.

15 The data for País Vasco are publicly available online. In the case of Cataluña the data were provided by the Autonomous Government upon request for this research.

16 This variable was obtained from the municipal database of CajaEspaña.

17 To compute this index, La Caixa takes into account the population, number of phones, automobiles, trucks and vans, banking offices and retail activities. In order to make this index comparable across municipalities we divided it by the population.

18 The raw data distinguishes between two destinations (job or school). Our variable is the arithmetic average of both. We also indicate that INE does not compute an index associated with these variables. Instead the report includes the percentage of people on seven intervals that go from "less than 10 min" to "more than 90 min". We took class marks in the intervals (90 for the last interval) and weighted each class mark by the percentage of population within the interval. The weighted sum can be interpreted as the average time employed to reach school or job and is the variable used in this paper.

19 Many of these variables (or similar indicators) are proposed by the EU Sponsorship Group on measuring social progress and by the OECD Better Life Index. For instance, the unemployment rate, excess mortality, quality of dwellings, overall level of education, air quality (PM_10_, O_3_), voluntary work, crime rates and voter turnout can be found in a very similar or identical form. There are also indicators which are similar to the ASC and commercial market share. Even though our selection of indicators is constrained by data availability, we believe it offers a close description of the QoL dimensions in a similar way as specified in those initiatives.

20 In a recent proposal, Vidoli et al. (2015) suggest the use of a directional distance function to introduce reasonable weight constrains.

21 DEA was developed to measure efficiency in production, where a set of inputs (resources) are combined to produce a set of outputs (products and services).

22 The political-administrative structure of Spain clusters municipalities into provinces and provinces into Autonomous Communities (ACs). There are 17 ACs and two autonomous cities (Ceuta and Melilla).

23 In order to provide this summary of the data, we first aggregated (arithmetic average) the two indicators within each of the eight dimensions for the municipality, then we computed the population weighted average of the dimension for each AC. This is done only for a description of the data in Table 4. In all the subsequent computations of the QoL scores, the 16 municipal level indicators are used independently and without any kind of previous aggregation.

24 Many of these are very small municipalities; the smallest has only three citizens registered.

25 In some ACs there is statistical information of per capita income at the municipal level; however, the methodologies of the regional statistical offices are not homogeneous and their data are not comparable.
